# The Effect of Toll-Like Receptor 4 on Macrophage Cytokines During Endotoxin Induced Uveitis

**DOI:** 10.3390/ijms13067508

**Published:** 2012-06-18

**Authors:** Shuo Yang, Hong Lu, Jing Wang, Xin Qi, Xuhui Liu, Xiaolong Zhang

**Affiliations:** Department of Ophthalmology, Chaoyang Hospital, Capital Medical University, No.8 Baijiazhuang Road, Chaoyang District, Beijing 100020, China; E-Mails: yangshuo@sohu.com (S.Y.); daxi666@163.com (J.W.); qixin1343@sina.com (X.Q.); knutliu@163.com (X.L.); lxxiaolong@yahoo.com.cn (X.Z.)

**Keywords:** Toll-like receptor 4, mice peritoneal macrophage, endotoxin-induced uveitis, cytokine

## Abstract

Toll-like receptor 4 (TLR4) signal activation of macrophages can lead to endotoxin-induced uveitis (EIU). Previously, our research group has demonstrated a higher expression of TLR4 *in vivo* during EIU than normal. In this study, we analyzed levels of peritoneal macrophage cytokines from C3H/HeN mice with LPS stimulation *in vitro* to elucidate the effect of TLR4 on cytokines during EIU.

## 1. Introduction

Uveitis is a common inflammatory eye disease. Acute anterior uveitis (AAU) is the most common form of uveitis which accounts for 50%–60% total number of uveitis cases [1]. Currently, uveitis is considered an autoimmune disease causing damage by the eyes’ normal immune function. However, the exact pathogenesis and initiating factor of uveitis are not yet clear. Studies suggest that Gram-negative bacterial infections may begin this process [2]. Lipopolysaccharide (LPS), a major component of G-bacterial cell walls, is a main starting factor of the innate immunity. It is Toll-like receptor-4 (TLR4), expressed in macrophages as a main specific LPS recognition and cellular activation signaling receptor that plays an important role in starting innate immune [3]. Our team has successfully established a model of anterior uveitis induced by vibrio cholera endotoxin in Wistar rats. There was a significantly higher expression of TLR4 in the experiment group compared with the control group. We also found TLR4 located mainly on the membrane of macrophages in the iris stromal layer. These studies indicate that TLR4 on macrophages may be involved in the activation of uveitis [4]. McMenamin [5] reported that the uveal tract in mice, as in rats, contains rich networks of resident tissue macrophages. The networks of resident tissue macrophages in the uveal tract of mice closely resemble those in the peritoneal cavity. To investigate the potential role of TLR4 in the pathogenesis of uveitis, we established C3H/HeN (wild type) mouse model, and cultured peritoneal macrophages *in vitro*. Then, cultured macrophages were stimulated with LPS and pre-treated with anti-TLR4 monoclonal antibodies (MTS 510). We previously reported changes of cytokines in the anterior chamber and plasma of C3H/HeN mice after LPS stimulation [6]. In this study, we cultured peritoneal macrophages from C3H/HeN mice and analyzed the concentrations of cytokines TNF-α, IFN-γ, IL-10, IL-6 and IL-1β in the supernatant after treatment with vibrio cholera endotoxin to show the important role of the TLR4 signaling pathway in the pathogenesis of uveitis *in vitro*, which can provide new ideas of blocking the upper way in the treatment of uveitis.

## 2. Results

### Clinical manifestation of endotoxin-induced uveitis (EIU)

**Figure 1:**
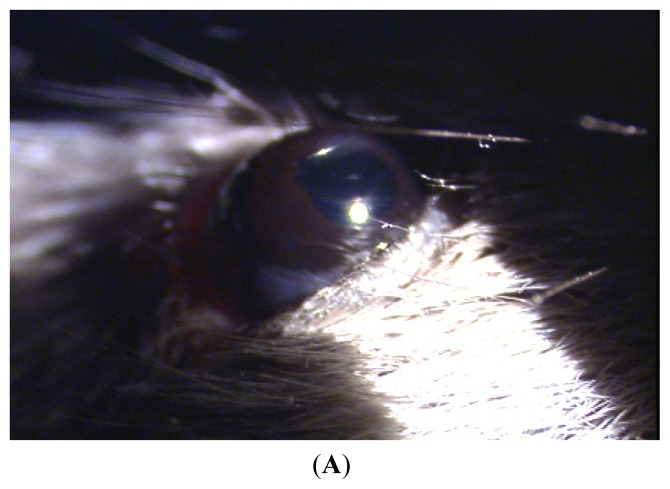

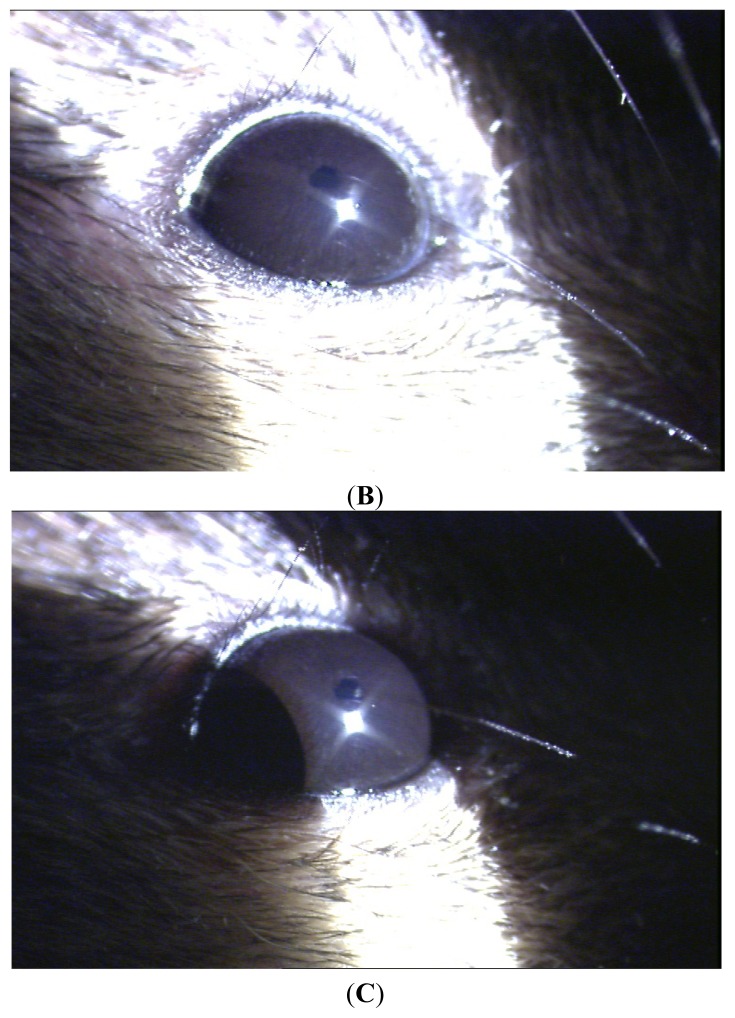
The clinical manifestation of C3H/HeN mice after injection of endotoxin. (**A**) Iris hyperemia, pupil adhesion after mydriasis with compound tropicamide in C3H/HeN mice at 24 h after injection of endotoxin; (**B**) No anterior segment inflammation in the control at 24 h after injection of endotoxin; (**C**) No anterior segment inflammation in the MTS510 group at 24 h after injection of endotoxin.

Acute anterior uveitis (AAU) was successfully induced in the C3H/HeN mice that were intraperitoneal-injected with 200 μg LPS. Twenty-four hours after injection, iris hyperemia, anterior chamber flare, fibrin filtration and other clinical symptoms were observed (Figure 1A). No anterior segment inflammation was observed in the MTS510 group or the control group after LPS injection (Figure 1B,C).

### Cell Identification

**Figure 2:**
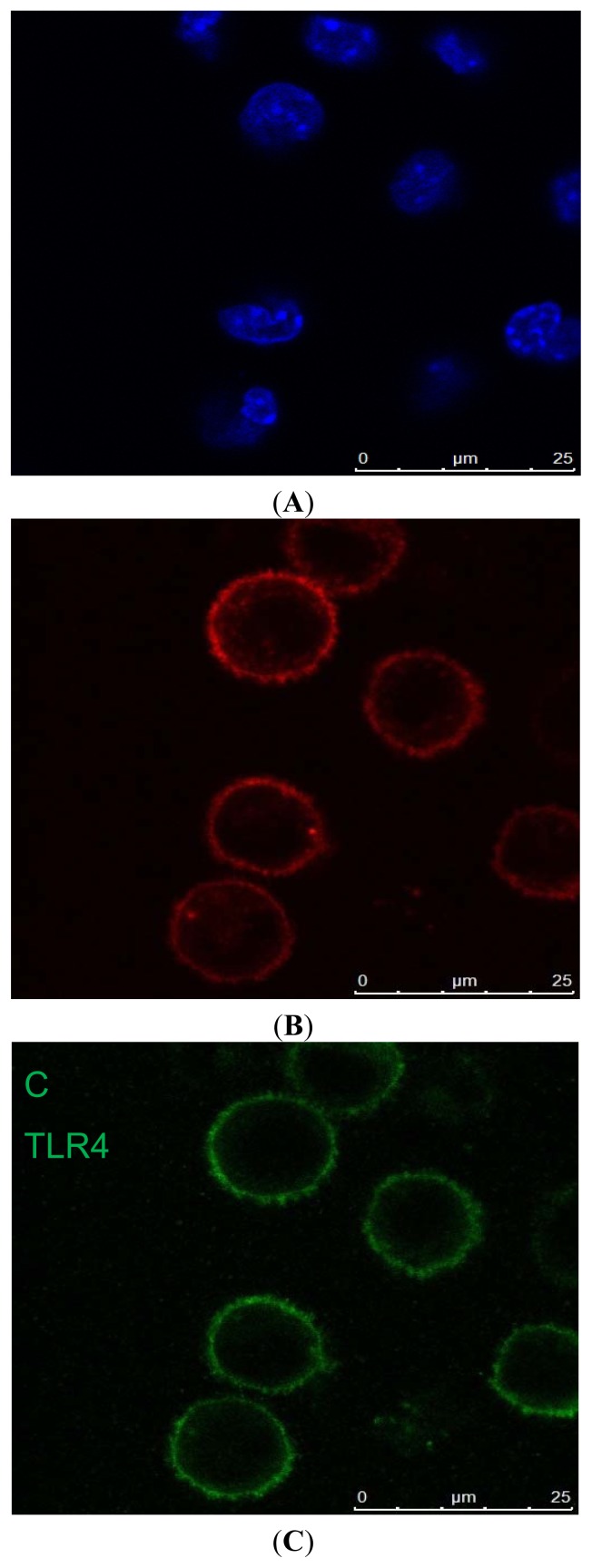
Immunohistochemical studies for Toll-like receptor-4 (TLR4) and F4/80. (**A**) No staining was seen in the negative control group; (**B**) C3H/HeN mouse peritoneal macrophages were marked with F4/80 staining. Cells were approximately round; (**C**) The TLR4+ cells of C3H/HeN mice possessed round-ovoid morphology, expressed in the membrane without lipopolysaccharide (LPS) stimulation.

Peritoneal macrophages; the nucleus of the cells was round, kidney-shaped, or irregular. F4/80 and Toll-like receptor 4 (TLR4) could not be detected in the negative group (Figure 2A). Mouse peritoneal macrophages were marked with F4/80 staining; cells were approximately round (Figure 2B). The TLR4+ cells of C3H/HeN mice (Figure 2C).

**Figure 3:**
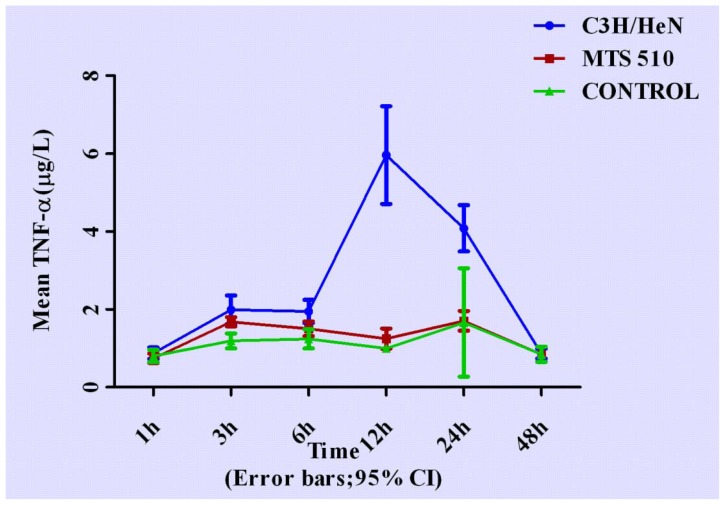
The expression levels TNF-α in the three groups at different times (expressed as the mean (mg/L) ± SD). Peritoneal macrophages of the three groups are treated with LPS. After stimulation, the concentration of TNF-α began to rise from 6 h and reached peak concentration at 12 h, then returned to its original level after 48 h in C3H/HeN group. The other two groups did not have obvious changes throughout the course.

**Figure 4:**
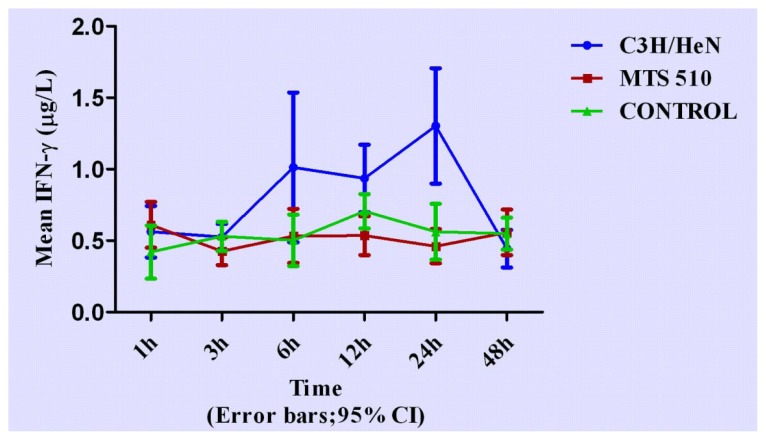
The expression levels IFN-γ in the three different groups at different time points (expressed as the mean (mg/L) ± SD). Peritoneal macrophages of the three groups are treated with LPS. After stimulation, the concentration of IFN-γ began to rise from 3 h, reaching peak concentration at 24 h and returned to its original level after 24 h in the C3H/HeN group. The other two groups did not have obvious changes throughout the course.

**Figure 5:**
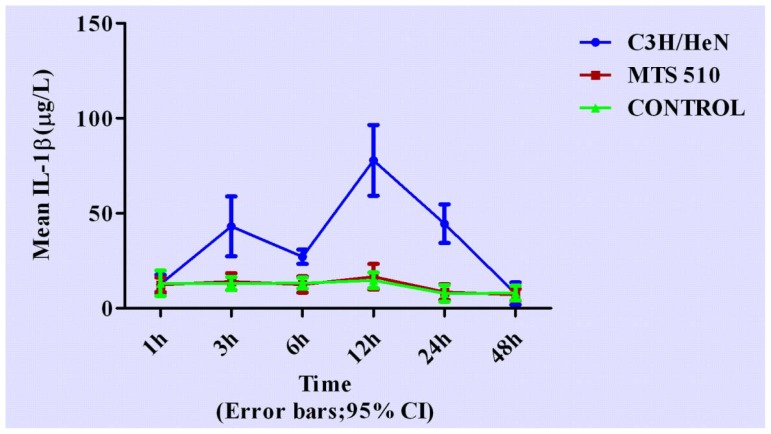
The expression levels IL-1β in the three different groups at different time points (expressed as the mean (mg/L) ± SD). Peritoneal macrophages of the three groups are treated with LPS. In the C3H/HeN group, the concentration of IL-1β rose in the early stage after LPS stimulation. Its peak concentration appeared at 3 h and 12 h. After 12 h, IL-1β concentration began to decrease and returned to its original level after 48 h. The other two groups did not have obvious changes throughout the course.

**Figure 6:**
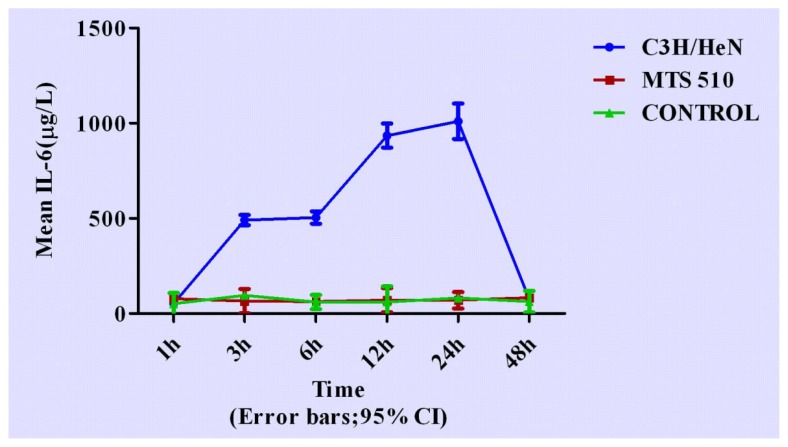
The expression levels IL-6 in the three different groups at different time points (expressed as the mean (mg/L) ± SD). Peritoneal macrophages of the three groups are treated with LPS. In the C3H/HeN group, the concentration of IL-6 rose to a high level from 3 h to 6 h after LPS stimulation, reaching a higher level from 12 h to 24 h and finally returning to its original level after 48 h. The peak concentration appeared at 24 h after LPS stimulation. The other two groups did not have obvious changes throughout the course.

**Figure 7:**
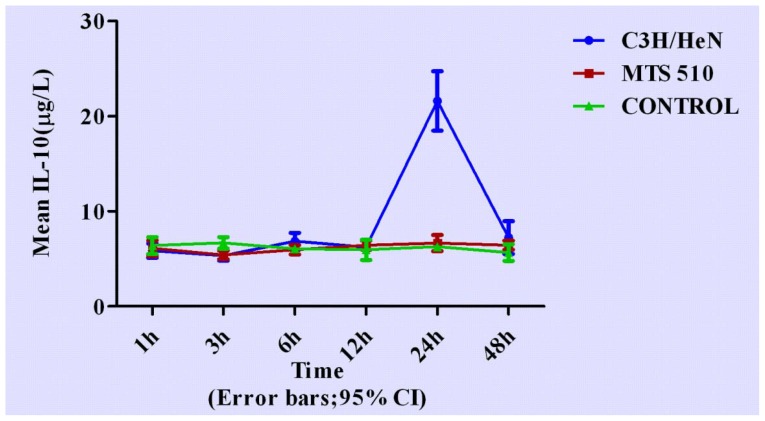
The expression levels IL-10 in the three different groups at different time points (expressed as the mean (mg/L) ± SD). Peritoneal macrophages of the three groups are treated with LPS. After stimulation, the concentration of IL-10 began to rise from 12 h, reaching peak concentration at 24 h and returning to its original level after 48 h in C3H/HeN group. The other two groups did not have obvious changes throughout the course.

Concentrations of cytokines at different time points are shown in Figures 3–7.

### 2.1. Concentration of Tumor Necrosis Factor-α in Groups of C3H/HeN, MTS 510 and CONTROL

After treatment with lipopolysaccharide (LPS), concentration of TNF-α rose rapidly from 6 h in the C3H/HeN group, reaching peak concentration at 12 h (*p* < 0.001, respectively). It gradually went down but was still higher than the other two groups at 24 h (*p* < 0.001, respectively), then finally returned to its original level after 48 h (*p* = 0.735 and *p* = 0.971, respectively) when compared with the other two groups.

### 2.2. Concentration of Interferon-γ in Groups of C3H/HeN, MTS 510 and CONTROL

Concentration of IFN-γ in the three different experiment groups was lower than those of other cytokines observed. After treated with lipopolysaccharide (LPS), concentration of interferon-γ in C3H/HeN group rose from 3 h and was higher than that of the other two groups at 6 h, 12 h and 24 h (*p* < 0.001, respectively). The peak concentration appeared at 24 h (*p* < 0.001, respectively) when compared with the other two groups. No statistically significant differences were found at any other time point among the three groups.

### 2.3. Concentration of Interleukin-1β in Groups of C3H/HeN, MTS 510 and CONTROL

We found two peak concentrations of IL-1β in C3H/HeN group at 3 h and 12 h (*p* < 0.001 and *p* < 0.001, respectively) when compared with the other two groups. After 12 h, concentration IL-1β in C3H/HeN group began to decrease and returned to its original level after 48 h (*p* = 0.816 and *p* = 0.635, respectively).

### 2.4. Concentration of Interleukin-6 in Groups of C3H/HeN, MTS 510 and CONTROL

Concentration of IL-6 in C3H/HeN group changed dramatically compared with the other two groups after LPS stimulation. In C3H/HeN group, concentration of IL-6 rose up to a high level from 3 h (*p* < 0.001, respectively) to 6 h (*p* < 0.001, respectively), and continued to rise up to a higher level from 12 h to 24 h (*p* < 0.001, respectively), finally returning to its original level after 48 h (*p* = 0.532 and *p* = 0.627, respectively) when compared with the other two groups. The peak concentration appeared at 24 h. No significant differences were found between CONTROL and MTS 510 group at any other time during the course.

### 2.5. Concentration of Interleukin-10 in Groups of C3H/HeN, MTS 510 and CONTROL

Concentration IL-10 changed later than other cytokines observed in C3H/HeN group. It began to rise after 12 h (*p* = 0.553 and *p* = 0.426, respectively), reaching peak concentration at 24 h (*p* < 0.001, respectively), returning to its original level after 48 h (*p* = 0.872 and *p* = 0.437, respectively). No significant differences were found at any other time among the three groups.

## 3. Discussion

Acute anterior uveitis is a common refractory eye disease with the potential threat to visual loss. Our earlier study was to build an acute anterior uveitis animal model and experiment the basis of membrane receptors. In this study, we further investigated the molecular mechanism of the signal transduction pathway of TLR4 during EIU pathogenesis. Acute anterior uveitis, especially HLA-B27-associated AAU, is a common noninfectious uveitis, but clinical and laboratory research have proven that gram-negative bacteria species, such as klebsiella, salmonella, yersinia, and shigella can trigger it [2]. LPS is part of the outer protein of the cell wall of gram-negative bacteria. Through the LPS-binding protein, CD14, toll like receptor 4, LPS is able to communicate with some host cells and regulates cytokines such as IL-1b, IL-6, IL-12, and TNF-a [7], the production by dendritic cells and macrophages. LPS was observed to up-regulate TLR4-MD-2 expression by peritoneal macrophages *in vitro* [8,9]. As an activator of macrophages, LPS can induce monocyte/macrophage differentiation, maturation and activation, and enhance the release of pro-inflammatory cytokines from monocyte/macrophages [10–12]. Our previous study revealed that activation of TLR4 on macrophages, using LPS stimulation, resulted in the activation of the transcriptional factor and nuclear factor-κB (NF-κB), via an immunostimulatory intracellular signaling pathway [4]. In our study, we found levels of cytokines in the C3H/HeN group rose with time after LPS stimulation, while those of the MTS 510 group and control group did not have obvious changes. And we also found there is no significant difference between the MTS 510 and control groups. Here we believe that the macrophages of MTS 510 mice had no response to LPS because of TLR4 blockage [13]. MTS 510, an anti-mouse TLR4 mAb, is reported to block LPS-induced NF-κB activation [14]. Akashi *et al*. [14] reported that TLR4-MD-2 was rapidly down-regulated on peritoneal macrophages treated with MTS510 mAb in the presence of LPS. Moreover, LPS-induced TNF-a production was profoundly inhibited by MTS510. We believe that the effect of MTS510 blocking this pathway may offer a new direction to future treatment.

Recent studies have shown that cytokines play an important role in the pathogenesis of AAU [15]. To investigate the role of cytokines in this pathogenesis, we measured the concentrations of TNF-α, IFN-γ, IL-10, IL-6 and IL-1β of cultured macrophages after treatment with LPS at various time points. In the C3H/HeN group, we found TNF-α and IL-1β rose 3 h after LPS stimulation and reached a peak at 12 h after LPS stimulation. We thought these two cytokines were important in the initiation of the inflammatory response. LPS can stimulate large amounts of TNF-α secretion by the monocyte-macrophage system. TNF-α can promote inflammatory cell aggregation, activation, release of inflammatory mediators and an increase of inflammation symptoms. It rises early in the inflammatory response and quickly reaches a peak. As a major media activating cytokine cascade, it can induce “secondary” cytokine production, such as IL-1β, IL-6 and IL-8. IL-1β as one of the pro-inflammatory factors secreted from activated macrophages can mediate inflammatory response [16]. IL-1β can help leukocyte in blood with migration, interaction and aggregation via vascular endothelial cell activation. In our study, we found two concentration peaks of IL-1β, which appeared at 3 h and 12 h. It indicated that IL-1β was associated with the early stage of inflammation. Furthermore, it is possible that the secretion of IL-1β was influenced by other cytokines. We also found IL-10 changed later than the other cytokines. IL-10 inhibits proliferation as well as cytokine synthesis of CD4+ T cells [17]. Macrophages, if conditioned by IL-10 or -4, have been proposed to down-regulate inflammation and act to balance pro-inflammatory and anti-inflammatory immune reactions [18]. Thus, we believed that IL-10 played an important anti-inflammatory role in the late stage of inflammation. Schreiber, T. *et al*. reported that IL-10 is not a strictly a Th2 cytokine, but it shares many features with them, and was thought to act as a “deactivator” of macrophages [19]. In our study, we found that the peak of inflammation was 24 h after LPS injection, then the inflammation reaction decreased. So, the cytokine concentration was consistent with the clinical manifestations. Many reports have demonstrated that treatment with cytokines, such as IL-10, IL-4, and the neutralizing antibody of TNF can aid in the treatment of the disease. Muzio *et al*. [20] found that IL-10 down-regulated the expression of TLR4, a receptor that mediates the innate immune response through the LPS signal transduction pathway. Matta Bharati *et al*. [21] reported that the expression levels of IL-10 and TGF-β2 increased, while levels of TNF-α, IFN-γ, and IL-2 decreased in a tolerance-induced EIU model. So we think that cytokines may play different roles (cascade release or feedback adjustment) in different phases of TLR4 signal transduction pathway activation.

## 4. Experimental Section

### 4.1. Animals

Adult male C3H/HeN mice (6–8 weeks old) were obtained from the Vital River Laboratory Animal Technology Co. Ltd (Beijing, China). All mice were housed in pathogen-free conditions in cycles of 12 h light/12 h dark with free access to food and water. *In vivo*, 15 mice (*n* = 5 per group) were used to establish the endotoxin-induced uveitis. The specimens included 90 mice (*n* = 30 per group) used for the *in vitro* experiment. Throughout this study, all procedures adhered to the Institute for Laboratory Animal Research Guidelines (Guide for the Care and Use of Laboratory Animals).

### 4.2. Experimental Groups

Animals were randomly divided into three groups: Control group, C3H/HeN group, MTS510 group (pre-treated with MTS 510). At 1 h, 3 h, 6 h, 12 h, 24 h and 48 h specimens were collected after mouse peritoneal macrophages were stimulated with LPS.

### 4.3. Establishing the EIU Model

The experimental group received an intraperitoneal injection of 200 μg vibrio cholera (classical Biotype, serotype Ogawa, provided by the Lanzhou Institute of Biologic Products Lanzhou, China) dissolved in 100 μL sterile saline (NS). The control group was intraperitoneally-injected with 100 μL saline solution. The eyes were examined using a slit microscope before injection and after several hours had elapsed.

### 4.4. Culture and LPS Stimulation of Peritoneal Macrophages

The mice were injected, intraperitoneally, with 2 mL of 3% thioglycollate (Taigemei, Biotechnology, Beijing, China). After four days, peritoneal cells were collected by lavage with an average viability of 98%. The cell viability was evaluated using the trypan (Sigma, St. Louis, MO, USA) blue exclusion test (0.4%). Cells were seeded onto 24-well plates (1 × 10^5^ cells/well) in RPMI 1640 medium (Hyclone, Logan, UT, USA), supplemented with 2 mM glutamine (Hyclone), antibiotics (100 U/mL of penicillin and 100 U/mL of streptomycin), and 10% heat-inactivated fetal bovine serum (Hyclone) for 24 h to allow the macrophages to adhere to the plates. Non-adherent cells were subsequently removed by washing with Hank’s balanced salt (HBSS) solution, confirmed with F4/80 stain. The adherent macrophages were grown in pre-placed coverslips in RPMI 1640 medium, containing 10% fetal bovine serum and antibiotics. *In vitro*, peritoneal macrophages of the LPS and MTS 510 groups were treated with 1 μg/mL vibrio cholera, while those of the control group were treated with PBS. Macrophages, in the presence or absence of LPS, were used for the experiments. The anti-TLR4 monoclonal antibody (rat monoclonal antibody; Santa Cruz Biotechnology, Santa Cruz, CA, USA) group, with adherent macrophages, was pretreated with anti-TLR4 monoclonal antibody (with a final concentration of 10 μg/mL) for 1 h, then washed, three times, with HBSS solution. Subsequent, identical steps were taken with the other groups.

### 4.5. Immunofluorescence

The adherent cells were washed with PBS, fixed in freshly prepared 4% paraformaldehyde in PBS for 15 min at room temperature, washed three-times, with PBS, permeabilized with HEPES-Triton buffer (20 mM HEPES, 300 mM sucrose, 50 mM NaCl, 3 mM MgCl_2_, 0.5% Triton X-100, pH 7.4) on crushed ice for 1 h, and then washed three times, with PBS. The cells were blocked with PBS containing 10% BSA for 1 h at room temperature, and incubated with F4/80 (rat monoclonal antibody; Santa Cruz Biotechnology) in a humidified chamber, at 4 °C overnight. (all antibodies 1:50 in 10% BSA/PBS). Excessive antibodies were removed by washing the coverslips three times, with PBS. The cells were incubated with fluorescein-conjugated goat anti-rabbit IgG, rhodamine-conjugated goat anti-rat IgG, and goat anti-mouse IgG (1:200 in PBS; Zhongshan Goldbridge Biotechnology, Beijing, China) for 2 h and were protected from light and room temperature. After being washed three times, with PBS, the cells were mounted onto a glass slide, using a mounting medium. Negative controls included replacing the first or second primary antibody with species- and isotype-matched irrelevant antibodies. Blank controls included replacing the first or second primary antibody with PBS. Slides were examined under a fluorescence microscope (Leica-DM-4000B; Leica, Wetzlar, Germany). Five high power fields were selected to analyze each stain by a single masked observer. Images were captured using an inverted confocal laser-scanning microscope (Leica-DM-IRE2; Leica).

### 4.6. ELISA

(Enzyme-linked immunosorbent assay for cytokines): tumor necrosis factor-α (TNF-α), interferon-γ (IFN-γ), interleukin-10 (IL-10), interleukin-6 (IL-6) and interleukin-1β (IL-1β) in the supernatant of cultured peritoneal macrophages were measured using a commercial mouse TNF-α, IFN-γ, IL-10, IL-6 and IL-1β enzyme-linked immunosorbent assay (ELISA) kit according to the manufacturer’s protocol. The absorbance was then detected at 450 nm to evaluate cytokine concentrations.

### 4.7. Data Processing and Statistical Analysis

Statistic analysis was performed using SPSS13.0 (SPSS Inc., Chicago, IL, USA) software. For multiple comparisons, different groups were analyzed using the one-way ANOVA technique, followed by Fisher’s Least Significant Difference Procedure (LSD) tests. A *p*-value, less than or equal to 0.05, was interpreted as indicating statistical significance when comparing different groups.

## 5. Conclusions

In summary, analyzing cytokine changes of the supernatant of cultured macrophages in different mice group after LPS stimulation, our results indicate the important role of TLR4, a macrophage trans-membrane protein receptor, during EIU, and help us understand cytokine changes from the beginning of this signal transduction pathway. TLR4 signal activation can lead to the cascading expansion effect of cytokines during EIU. Further investigation into the blockage of this pathway may provide more effective treatment for acute anterior uveitis.
